# Feasibility of the pupillary pain index as a guide for depth of analgesia during opioid-sparing anesthesia with continuous infusion of dexmedetomidine

**DOI:** 10.1186/s44158-023-00112-8

**Published:** 2023-08-14

**Authors:** Martino Stefanini, Elena Cagnazzi, Stefano Calza, Nicola Latronico, Francesco A. Rasulo

**Affiliations:** 1 Department of Medical and Surgical Specialties, Radiological Sciences and Public Health, University of Brescia, 25123 Brescia, Italy; 2 Department of Anesthesia, Intensive Care and Emergency, ASST Spedali Civili University Hospital, Piazzale Spedali Civili, 1, 25123 Brescia, Italy; 3 Unit of Biostatistics and Bioinformatics, Department of Molecular and Translational Medicine, University of Brescia, 25123 Brescia, Italy

**Keywords:** Dexmedetomidine, Pupil, Nociception, Pupillary reflex, Analgesia

## Abstract

**Background:**

The pupillary dilation reflex (PDR) is an objective indicator of analgesic levels in anesthetized patients. Through measurement of the PDR during increasing tetanic stimulation (10–60 mA), it is possible to obtain the pupillary pain index (PPI), a score that assesses the level of analgesia.

**Objectives:**

The depth of analgesia during opioid-sparing anesthesia (OSA) with continuous infusion of dexmedetomidine in addition to general anesthesia was assessed.

**Design:**

Observational prospective feasibility pilot study

**Setting:**

This study was performed in the operating rooms of the Spedali Civili University-affiliated hospital of Brescia, Italy.

**Patients:**

Forty-five adults who underwent elective open (5-cm incision) surgery under general anesthesia (78% inhalation anesthesia), from Feb. 18th to Aug. 1st, 2019, were enrolled. Exclusion criteria were as follows: implanted pacemaker or ICD, ophthalmological comorbidities, chronic opioid use, peripheral neuropathy, other adjuvant drugs, epidural analgesia, or locoregional block.

**Main outcome measures:**

The first aim was to verify the feasibility of applying a study protocol to evaluate the depth of analgesia during intraoperative dexmedetomidine administration using an instrumental pupillary evaluation. The secondary outcome was to evaluate appropriate analgesia, drug dosage, anesthesia depth, heart rate, blood pressure, transient side effects, postoperative nausea and vomiting (PONV), and pain numerical rating scale (NRS) score.

**Results:**

Thirty out of 50 patients (60%) treated with dexmedetomidine during the study period were included in the DEX group (8 males, age 42 ± 13 years, BMI 45 ± 8), and 15 other patients were included in the N-DEX group (8 males, age 62 ± 13 years, BMI 26 ± 6). Patients who underwent bariatric, abdominal, or plastic surgery were enrolled. At least 3 pupillary evaluations were taken for each patient. *PPI* ≤ 3 was observed in 97% of patients in the DEX group and 53% in the N-DEX group. Additionally, the DEX group received less than half the remifentanil dose than the N-DEX group (0.13 ± 0.07 vs 0.3 ± 0.11 mcg kg^−1^ min^−1^). The average dose of dexmedetomidine administered was 0.17 ± 0.08 mcg kg^−1^ h^−1^.

**Conclusion:**

The feasibility of applying the protocol was verified. An OSA strategy involving dexmedetomidine may be associated with improved analgesic stability: a randomized controlled trial is necessary to verify this hypothesis.

**Trial registration:**

Trial.gov registration number: NCT05785273

## Key points

• Pupillary pain index is an objective measure of analgesic level in anesthetized patients.

• A pupillometer and pupillary pain index may be used after a brief training period.

• A constant rate infusion of dexmedetomidine, as an adjuvant during general anesthesia, may improve and stabilize analgesia.

• The use of pupillary pain index is a way to reduce opioid administration and maintain adequate analgesia.

## Background

During surgery, opioids are commonly used to maintain optimal analgesic coverage. Despite their efficacy and widespread use, opioids are associated with several postoperative side effects, like prolonged sedation, delayed weaning from mechanical ventilation, respiratory failure, delirium, hyperalgesia, shivering, postoperative nausea and vomiting (PONV), urine retention, and opioid abuse [1].

For this reason, in the past years, many strategies have been tested to reduce opioid use during the perioperative period, such as opioid-free anesthesia (OFA) or opioid-sparing anesthesia (OSA). These techniques can reduce opioid-related side effects with equivalent intraoperative analgesia compared to opioid-based anesthesia [2, 3]. Moreover, patient satisfaction seemed to increase with OFA [4]. OFA combines several therapeutic strategies, including regional anesthesia and administration of adjuvants as well as lidocaine, ketamine, magnesium, and α2 agonists.

Dexmedetomidine is a highly selective α2 agonist, with sedative and pain-relieving effects, and its perioperative use reduces intraoperative opioid, as well as hypnotics, consumption [5, 6]. Several studies have reported also a decrease in postoperative pain intensity, postoperative opioid use, and postoperative adverse events (PONV, delirium, agitation, and shivering) [7]. These features are particularly advantageous in bariatric patients [8].

Coeckelenbergh et al. assessed the maintenance of an appropriate analgesic level during general anesthesia, with the infusion of dexmedetomidine and remifentanil guided by the “nociception level index” (NoL) [9]. No other studies on analgesic stability during dexmedetomidine-remifentanil infusion have been detected by our group so far.

Traditionally, during general anesthesia, the level of nociception is indirectly assessed by observing heart rate (HR), blood pressure (BP), and considering also the surgical step. Recently, new indicators have been proposed to obtain objective and standardized nociception assessment such as HR variability, plethysmography wave amplitude, skin conductance level, processed EEG, and pupillary reflex [10, 11]. The pupillary diameter increases in response to nociceptive stimuli in both awake and anesthetized patients (pupillary dilation reflex (PDR)).

Some studies have shown that PDR can be related to both painful stimulation [12] and analgesic depth [13], but not to neuromuscular blockade [14] or hypnotic drug administration [12]. PDR was used in a randomized controlled pilot study as a guide for the management of remifentanil, with a reduction in intraoperative and postoperative opioid use [15].

Recently, another rating scale for nociception has been proposed: pupillary pain index (PPI). This index is based on increasing intensity tetanic stimulation, delivered by two skin electrodes placed on the forearm. PPI evaluates PDR from 1 to 9, for each stimulus, and is obtained according to the intensity of the stimulation needed to reach the threshold of *PDR* > 13%. A PPI of 1 to 3, 4 to 6, or 7 to 9 describes numbed PDR and deep analgesia, medium PDR and suboptimal analgesia, and highly reactive PDR and uncontrolled pain, respectively. A decrease in the use of opioids was detected when this score was used as a criterion during intraoperative administration of opioids [16–18].

This study evaluated the feasibility of measuring the analgesic depth during OSA, with continuous infusion of dexmedetomidine and remifentanil, using PPI as an indicator of nociception.

## Materials and methods

Ethics approval for this observational prospective pilot study (Comitato Etico Provinciale. Provincia di Brescia, Italy. Ref. NP 3675) was provided by the local ethics committee, Italy (Chairperson Prof. S. Sigala).

This study was conducted at the Spedali Civili University-affiliated hospital in Brescia, Italy. Written informed consent was obtained from all the study participants. Due to the pilot nature of this study, an estimation of the size sample was not done [19].

### Outcomes

The primary outcome was to verify the feasibility of assessing the depth of analgesia during dexmedetomidine administration. As a measure of feasibility, we considered the number of pupillary measurements performed after incision and the percentage of patients in whom PONV and NRS were evaluated.

The secondary outcome was to assess the depth of intraoperative analgesia with the PPI score, the total consumption of remifentanil and dexmedetomidine, the depth of anesthesia with bispectral index (BIS) or patient state index (PSI), the incidence of side effects, PONV, and postoperative intolerable pain.

### Inclusion and exclusion criteria

Patients aged 18 years and older, who underwent open surgery involving at least one surgical incision of more than 5 cm and with remifentanil as an intravenous analgesic, were included.

Patients presenting with an implanted pacemaker or ICD, ophthalmological comorbidities, chronic opioid use, peripheral neuropathy, continuous infusion of adjuvant drugs other than dexmedetomidine, and epidural analgesia or locoregional block were excluded.

- DEX group: All bariatric patients, scheduled for mini-laparotomic gastric bypass and restrictive gastroplasty.
- N-DEX group: Patients scheduled for any kind of surgery with a surgical incision of 5 cm at least and without locoregional anesthesia or adjuvants.

### Study protocol

#### General considerations on anesthesia management

All patients received a standard anesthesia protocol, which was uninfluenced by this study. Considering the observational nature of the study, the anesthesiologists who participated were not asked to modify their anesthesiological conduct. The only intervention permitted was an increase in remifentanil infusion if a PPI compatible with uncontrolled pain was detected. The anesthetic protocol for bariatric patients included the use of an inhalatory hypnotic agent, preferably desflurane, and remifentanil. Generally, the anesthesiologists have to adhere to this “bariatric protocol,” while they could choose the hypnotic agent for other kinds of patients.

#### Study cases

Patients treated with dexmedetomidine combined with remifentanil in continuous infusion were included in the DEX group. Patients who underwent general anesthesia (inhaled or intravenous general anesthesia with continuous infusion of remifentanil) without adjuvants were included in the N-DEX group.

Patients in the DEX group received a dexmedetomidine loading dose of 0.5–1 μg/kg (ADJUSTED weight) over 15 min. After intubation, they received general anesthesia with desflurane or sevoflurane (MAC 0.6–1, according to BIS or Psi); dexmedetomidine (0.2–0.4 μg/kg/h (lean body weight (LBW)), with constant infusion rate unless hypotension or bradycardia not responsive to standard treatments (fluids, atropine, ephedrine, ethylephrine) occurred; and remifentanil (0.02–0.2 μg/kg/min (LBW)) based on clinical and instrumental assessment (HR, BP, BIS, or PSI).

Patients in the N-DEX group received premedication with benzodiazepines according to the assessment of the anesthesiologist and the condition of the patient. After intubation, general anesthesia was administrated with inhaled (desflurane or sevoflurane; MAC 0.6–1) or intravenous (propofol) anesthetics (according to BIS or Psi) and remifentanil (0.02–0.2 μg/kg/min (LBW) based on clinical and instrumental assessment (HR, BP, BIS, or PSI).

#### Pupil evaluation timepoints

A baseline pupil evaluation was performed between the induction of general anesthesia and the onset of surgery. After the incision, at least three pupil evaluations were performed; the first was taken 15 min after the incision followed by the subsequent measurements which were 15 min apart and taken at least 10 min after any change in dosage. HR, BP, depth of anesthesia (with BIS or Psi monitoring), and rate of perfusion of remifentanil and dexmedetomidine were recorded. HR, BP, electrocardiogram, peripheral oxygen saturation, and BIS were monitored continuously or at least (for noninvasive BP) every 5 min; the values were recorded every 5 min. The anesthesia management did not change because of pupil assessments unless a PPI consistent with uncontrolled pain was detected. In these cases, remifentanil infusion was increased. In all other cases, we aimed to use the lowest remifentanil infusion rate for normal blood pressure, HR, and BIS.

LBW, calculated using the Janmahasatian formula [20], was used to evaluate remifentanil and dexmedetomidine consumption per kilogram. Actual body weight (ABW) was used to calculate propofol consumption per kilogram.

During anesthesia, all possible side effects and treatments were registered; after waking from anesthesia, PONV and intolerable pain (considered as an *NRS* > 4, as suggested by Gebershagen H. et al.) were assessed [21].

### Pupillary measurements

To evaluate the depth of analgesia, PPI was measured using an AlgiScan pupillometer (IDMED, Marseille, France). Using the incorporated PPI protocol, this device can send increasing tetanic stimulation (from 10 to 60 MA, 100 Hz) via two cutaneous electrodes placed along the forearm while simultaneously measuring pupillary dilation. At each stimulation, the pupillometer measured pupillary dilation; when the dilation exceeded 13% of the basal diameter, the stimulation stopped, and the PPI score is calculated. PPI score is incremented by 1 point if pupil dilation is above 20%. Table 1 shows the PPI scoring algorithm used in this study.

**Table 1:** PPI score

Maximum stimulation intensity (mA)	Pupillary reactivity	PPI score
10	Pupil dilates > 13% after a stimulation of 10 mA	9
20	Pupil dilates > 13% after a stimulation of 20 mA	8
30	Pupil dilates > 13% after a stimulation of 30 mA	7
40	Pupil dilates > 13% after a stimulation of 40 mA	6
50	Pupil dilates > 13% after a stimulation of 50 mA	5
60 (1st stimulation)	Pupil dilates > 13% after the 1st stimulation of 60 mA	4
60 (2nd stimulation)	Pupil dilates > 13% after the 2nd stimulation of 60 mA	3
60 (2nd stimulation)	Pupil dilates < 13%, but > 5%, after the 2nd stimulation of 60 mA	2
60 (2nd stimulation)	Pupil dilates < 5% after the 2nd stimulation of 60 mA	1

### Data presentation

Data were collected and analyzed using Microsoft Excel 2011. Given the pilot nature of this study, only descriptive statistics were reported [19].

Continuous variables are expressed as means and standard deviations (SD), and discrete variables are expressed as counts (and percentages).

## Results

We included 45 patients scheduled for surgery from the 19th of February to the 1st of August 2019; 30 were included in the DEX group and 15 in the “N DEX” group. Twenty-four patients treated with dexmedetomidine were not enrolled due to operator or device unavailability.

All patients in the DEX group received general anesthesia plus dexmedetomidine, while those in the N-DEX group received general anesthesia without dexmedetomidine. Remifentanil was the drug of choice for surgical analgesia in both groups. Patient characteristics can be found in Table 2.

**Table 2:** Patient’s characteristics

	TOT (*n* = 45)	“DEX” (*n* = 30)	“N-DEX” (*n* = 15)
Gender			
Male	16 (36%)	8 (27%)	8 (53%)
Female	29 (64%)	22 (73%)	7 (47%)
Age (years)	48 (*SD* 16)	42(*SD* 14)	62 (*SD* 13)
Weight (kg)	106.8 (*SD* 34.4)	125.5 (*SD* 27)	73.2 (*SD* 20.1)
Height (m)	1.67 (*SD* 0.08)	1.65 (*SD* 0.07)	1.69 (*SD* 0.11)
BMI (kg/m^2^)	38.5 (*SD* 119)	45 (*SD* 8)	25.5 (*SD* 5.8)
ASA score			
I [num]	1 (10%)	0 (0%)	1 (7%)
II [num]	10 (22%)	3 (10%)	7 (47%)
III [num]	32 (71%)	27 (90%)	5 (33%)
IV [num]	2 (4%)	0 (0%)	2 (13%)

### Surgical procedures

In the DEX group, 29 (97 %) patients underwent mini-laparotomic bariatric surgery, one of whom underwent another kind of abdominal surgery. In the N-DEX group, 6 patients underwent abdominal surgery (40%), 6 mammary surgery (47%), and 2 large excisions for melanoma (13%).

### Feasibility

We were only able to include 30 out of 50 (60%) patients treated with dexmedetomidine during the study period due to the limited availability of resources. Obtaining at least three intraoperative PPI measurements per patient was considered as an indicator of feasibility. This indicator was met in all of the patients, for whom 3–5 measurements were obtained. NRS was assessed in 39 out of 45 (86%) patients and PONV in 41 out of 45 patients (91%).

### Intraoperative pupillary measurements

PPI was maintained ≤ 3 in every post-incision measurement in 29 out of the 30 patients (97%) in the DEX group. Considering the PPI measurement in this group, only one out of 124 PPI was above 3. Considering PPI measurement in the N-DEX group, PPI was less than or equal to 3 at every measurement only for 8 patients (53%); PPI was between 4 and 6 in 7 patients. No measurement was higher or equal to 7. Mean HR and BP and depth of anesthesia at the time of pupil evaluation are shown in Table 3.

**Table 3:** Heart rate (HR), mean blood pressure (BP), and depth of anesthesia at the time of pupil evaluation

	**TOT (*n* = 178)**	**“DEX” (*n* = 124)**	**“N-DEX” (*n* = 54)**
Depth of anesthesia			
Superficial [num] (*BIS* > 60; Psi > 50)	0 (0%)	0 (0%)	0 (0%)
Not superficial [num] (*BIS* < 60; Psi < 50	146 (82%)	94 (76%)	52 (96%)
Optimal [num] (*BIS* 40–60; Psi 20–50)	93 (52%)	53 (43%)	40 (74%)
Deep [num] (*BIS* < 40; Psi < 20)	53 (30%)	41 (33%)	12 (22%)
Not evaluable [num]	32 (18%)	30 (24%)	2 (4%)
MAP [mmHg] (SD)	69 (25)	77 (16)	62 (29)
HR [bpm] (SD)	71 (16)	70 (12)	72 (20)

### Side effects

Side effects were identified in 8 out of 30 (26.6%) and 8 out of 15 (46.7%) patients in the DEX and in the N-DEX groups, respectively. Hypotension was observed in 6 (20%) and bradycardia in 2 (6.6%) patients of the DEX group. A reduction in remifentanil dosage in two patients and a decrease in both (remifentanil and dexmedetomidine) in two patients were required. Two other (25%) patients received vasoconstrictors. Hypotension was recorded in 7 patients (46.7%) in the N-DEX group. Five of these patients were treated with a vasoconstrictor. An episode of light anesthesia was clinically detected in 1 (6.7%) patient in the N-DEX group, requiring the administration of an additional dose of propofol. No episodes of light anesthesia were detected in the DEX group.

### Drugs consumption

The intraoperative consumption of remifentanil was 0.13 (*SD* 0.07) and 0.30 (*SD* 0.11) μg/kg/min in the DEX and N-DEX groups, respectively. The mean MAC for inhalator agents was 0.8 (*SD* 0.13) and 0.86 (*SD* 0.17) in the DEX and N DEX groups, respectively. The observed intraoperative consumption of dexmedetomidine amounted to 0.17 (0.08) μg/kg/h (excluding the loading dose). Table 4 reassumes drug doses.

**Table 4:** Drugs doses

	**TOT (*n* = 45)**	**“DEX” (*n* = 30)**	**“N-DEX” (*n* = 15)**
Induction			
Propofol (SD) [mg]	218 (63)	236 (53)	150 (54)
Fentanyl (SD) [μg]	146.25 (55.9)	145 (56.2)	161 (57.7)
Succinylcholine	11 (24%)	11 (37%)	0 (0%)
Dose (SD) [mg]	115 (23)	115 (23)	0
Maintenance			
Rocuronium [num]	37 (82%)	30 (100%)	7 (47%)
Dose (SD) [mg]	70 (21)	71 (21)	61.7 (17.2)
Desfluorane [num]	24 (53%)	22 (73%)	2 (13%)
Sevofluorane [num]	11 (24%)	8 (27%)	3 (20%)
Dose (SD) [MAC]		0.8 (0.13)	0.86 (0.17)
Propofol [num]	10 (22%)	0 (0%)	10 (67%)
Dose (SD) [mg/kg/h]			6.57 (1.92)

### Post operative

PONV was assessed in 41 out of the 45 patients (28 with DEX and 13 with N-DEX), and the incidence was 12% (5 of 41), all of whom pertained to the “DEX” group. NRS for pain was evaluated in 39 out of the 45 patients, 27 in the DEX group and 12 in the N-DEX group. The incidence of intolerable pain (*NRS* > 4, as suggested by Gebershagen H. et al.) [20] was 20% (6 patients) in the DEX group and 23% (3 patients) in the N-DEX group.

## Discussion

The first aim of this study was to verify the feasibility of applying a protocol to evaluate the depth of analgesia during intraoperative dexmedetomidine administration using instrumental pupillary evaluation. The percentage of enrolled patients out of the total number of enrollable patients (treated with continuous infusion of dexmedetomidine) and the number of intraoperative pupillary evaluation (with a minimum of 3 detections) were chosen as feasibility indicators.

Daily clinical practice was not affected by the study-linked activities, since an additional operator was dedicated to data collection. The protocol for the use of dexmedetomidine in bariatric patients was introduced before the study was designed. Bariatric patients were chosen as “cases” being the only ones given dexmedetomidine as part of an anesthetic protocol.

Most patients undergoing similar abdominal surgeries were given other types of adjuvants (like lidocaine) or locoregional anesthesia, or they were urgent cases. This was an obstacle in enrolling additional cases to assess with the pupillometer.

Due to limited resources (research staff or equipment unavailability), the percentage of enrolled patients was 60% of all patients treated with dexmedetomidine during the study period. To increase the percentage of enrolled patients for a future randomized controlled trial, a preliminary training phase for anesthesiologists would be required.

Three to five measurements were performed intraoperatively on each patient, meeting the feasibility criterion. Collected data showed a halved average remifentanil consumption and a more stable depth of analgesia in the DEX group than in the N-DEX group. Remifentanil infusion was, in fact, 0.3 μg/kg/min and 0.13 μg/kg/min (LBW) in DEX and N-DEX groups, respectively. PPI measurements were ≤ 3 in 29 out of 30 patients and 8 out of 15 patients in the DEX group and N-DEX group, respectively.

Evaluating nociception using the PPI index, the DEX group showed better analgesic stability despite lower remifentanil infusion. This is the first study, to the knowledge of our group, to assess the depth of analgesia in patients receiving dexmedetomidine using PPI. Coeckelenbergh et al. [9] assessed analgesic depth using NOL technology, stating that dexmedetomidine has an opioid-sparing effect. The opioid-sparing effect, obtained with constant dexmedetomidine infusion during general anesthesia, is well reported in the literature. Some studies reported a lower opioid requirement during clinical-based opioid administration; nevertheless, they do not use objective scores to assess the depth of analgesia [22–24].

This study evaluated an existing clinical OSA protocol using PPI technology. The attempt was to determine the correct depth of analgesia during its “daily” management with dexmedetomidine, by adding nociceptive monitoring. Nociception was evaluated through pupillary reflex since it seemed to be less influenced by haemodynamic and cardiac variations than other monitoring methods.

### Limitations

Pupillary nociception monitoring has some limitations, such as noncontinuous detection, which requires clinician intervention. Free access to the patient’s head is also required, thereby excluding the possibility of applying the protocol to patients undergoing head and neck surgery.

In the postoperative period, an increased incidence of PONV was detected in the “DEX” group, unlike what had been reported in several trials and meta-analyses [6, 25]. This difference was expected because of the antiemetic effect of propofol in the N-DEX group and the prevalent type of surgery in the DEX group (gastric surgery). Further limitations to the consistency of the study include anthropometric differences between the two populations, about BMI, and differences in anesthesia protocols.

For this reason, besides the nature of the study, the results can only suggest some hypotheses to be proved. The PPI evaluations in this study highlighted possible insufficient analgesia in the control group, suggesting that nociception monitoring may play an important role in providing adequate and customized anesthesia. Furthermore, dexmedetomidine administration could be associated with a decrease in intraoperative remifentanil consumption and a more stable intraoperative anti-nociception coverage. These results require further confirmation and validation through larger, randomized, and controlled trials.

## Data Availability

The datasets used and/or analyzed during the current study are available from the corresponding author upon reasonable request.
